# *Ganoderma lucidum* Immune Modulator Protein rLZ-8 Could Prevent and Reverse Bone Loss in Glucocorticoids-Induced Osteoporosis Rat Model

**DOI:** 10.3389/fphar.2020.00731

**Published:** 2020-05-19

**Authors:** Yong Yang, Tian Yu, Huan Tang, Zhihui Ren, Qianwen Li, Juan Jia, Hongyu Chen, Jun Fu, Shengchen Ding, Qiang Hao, Dan Xu, Liping Song, Bo Sun, Fei Sun, Jin Pei

**Affiliations:** School of Pharmaceutical Sciences, Jilin University, Changchun, China

**Keywords:** rLZ-8, *Ganoderma lucidum*, osteoporosis, dexamethasone, glucocorticoids-induced osteoporosis (GIOP)

## Abstract

Immune modulation has been recognized as an effective anti-osteoporosis strategy since the pivotal role of the RANK/RANKL/OPG signaling in bone metabolism and remodeling was discovered. To investigate the potential preventive and/or therapeutic effects of immune modulator protein Ling Zhi-8 (LZ-8) on osteoporosis, the osteoporosis animal model was established in Wistar rats by intramuscular injection of dexamethasone (DEX), namely glucocorticoids-induced osteoporosis (GIOP) rat model. To investigate the potential preventive effect of rLZ-8 on GIOP, we co-treated the rats with DEX and rLZ-8 intraperitoneally during the GIOP modeling stage and analyze the bone mass measured by bone mineral content (BMC) and bone mineral density (BMD), as well as levels of phosphorus (Pi), calcium (Ca^2+^) and hydroxyproline (HOP) in femur of GIOP rats. Consistently, all results suggested that rLZ-8 could prevent bone loss in the femurs of GIOP rats. Through analyzing the trabeculae morphology and the trabeculae amount by H&E staining, we found rLZ-8 could also improve the structural deterioration in femurs of GIOP rats. In order to further verify the results and its mechanism obtained from bone analysis, multiple biomarkers, including minerals metabolism (Pi and Ca^2+^), bone formation markers (osteocalcin, ALP and IGF-1), bone resorption markers (TRACP5b, CTX-1 and HOP), cytokines (IL-1β, IL-6 and TNF-α), oxidative stress indicators (GSH-px, SOD and MDA) and hormone molecules (testosterone, estradiol, calcitonin and parathyroid hormone) have been detected in serum or urine of rats. Results of these biomarkers in serum or urine confirmed rLZ-8’s protective effect in GIOP. Through analyzing the relative expression level of OPG and RANKL in femurs *via* western blot, we foundrLZ-8 could increase OPG/RANKL ratio which could impede osteoclastogenesis process. To test the potential therapeutic effect of rLZ-8 on successfully generated GIOP rats, we administrated rLZ-8 to rats for three weeks starting from the ending day of 7 weeks treatment of DEX. We found rLZ-8 could also reverse the bone loss in GIOP rats. Through the BWs and organ coefficient analysis, we found rLZ-8 has little toxicity to the rats. Our results suggested that rLZ-8 may be developed into promising anti-osteoporosis drug with both preventive and therapeutic properties.

## Introduction

Glucocorticoids (GCs) have been widely prescribed as effective immunosuppressive agents in the treatment of allergies, inflammatory conditions, or cancer for decades (Vandewalle et al., 2018). However, prolonged use of GCs could impair the metabolic balance of bone and induce secondary osteoporosis termed as GCs-induced osteoporosis (GIOP) (Buckley and Humphrey, 2018; Wang et al., 2019). Currently, the most widely used anti-osteoporosis medications are anti-resorptive drugs, such as bisphosphonates (alendronate, risedronate, zoledronate, and ibandronate) and RANKL antibodies which are largely limited by the adverse events in high risk of gastrointestinal disturbance and infection respectively (Lorentzon, 2019). Recently, two novel anabolic drugs improving bone formation, teriparatide and romosozumab, which are recombinant human parathyroid hormone and sclerostin monoclonal antibody respectively, provide promising options for osteoporosis treatment with side effects in high risk of transient or persistent hypercalcemia and stroke (Saag et al., 2017; Peugh et al., 2019). Therefore, exploring effective agents from natural products with minimal side effects is one of the promising alternative strategies (An et al., 2016).

*Ganoderma* species (Ling Zhi or Reishi is a traditional Chinese medicine with more than 400 bioactive molecules used to treat various medical conditions including tumor, infection, aging, immunological disorders and so on (Cor et al., 2018). Taxon name of *Ganoderma lucidum* has widely been used for most *Ganoderma* species for years, while the species authenticity has been challenged by analyzing the species-specific DNA sequence in recent years (Loyd et al., 2018) (Caution: we reserved the taxon name of *G. lucidum* as the original literature throughout this paper). Extracts of *G. lucidum* have been reported to improve the bone loss of ovariectomized (Ovx) rats significantly, and are likely through regulating osteoclastogenesis by compounds like ganoderic acid DM and ganomycin I (Miyamoto et al., 2009; Tran et al., 2018). Ling Zhi-8 (LZ-8), the first fungal immunomodulatory protein (FIP) family protein isolated from *G. lucidum in* 1989 (Kino et al., 1989), acts as an effective immune modulator through stimulating the expression of various cytokines like IL-2, TNF-α, IL-6 and so on (Haak-Frendscho et al., 1993; Liang et al., 2009; Hsu et al., 2013; Wu et al., 2015). Recombinant LZ-8 (rLZ-8) expressed in *Pichia pastoris* strain GS115 exhibited comparable capacity as native LZ-8 purified from *G. lucidum in vivo* (Xue et al., 2008; Huang et al., 2009; Liang et al., 2009; Liang et al., 2012). Considering that GIOP is caused by disorder of immune regulation by GCs prolonged use, we are very interested in investigating whether LZ-8, the immune modulator derived from *G. lucidum*, has preventive and/or therapeutic effects in treating GIOP.

## Materials and Methods

### Materials

Dexamethasone (DEX) sodium phosphate injection (2 mg/ml) (H41020327, Henan) was obtained from Runhong Pharmaceutical Company. Alendronate sodium (AS) tablets (H20083481, Hainan) were purchased from Aventis pharmacy. The tablets were smashed into powders and dissolved in normal saline with a concentration of 0.25 mg/ml as stock solution. rLZ-8 expressed in *P. pastoris* was obtained from Drilling Research Center of Jilin University as described (Xue et al., 2008; Huang et al., 2009; Liang et al., 2009; Liang et al., 2012). The stock solution of rLz-8 is 1.188 mg/ml and is further diluted into 45.74 μg/ml, 22.87 μg/ml and 11.43 μg/ml by saline solution immediately before use. Anti-OPG (bs-0431R, Beijing) and anti-RANKL/CD254 (bs-0747R, Beijing) were purchased from Bioss Antibodies Company. Alkaline Phosphatase (ALP) detection kit (A059-2, Nanjing), calcium (Ca^2+^) detection kit (C004-2, Nanjing), phosphorus (Pi) detection kit (C006-3, Nanjing), tartaric-acid-resistant acid phosphatase (TRACP5b) detection kit (A058-2, Nanjing), superoxide dismutase (SOD) detection kit (A001-3, Nanjing), malonaldehyde (MDA) detection kit (A003-3, Nanjing), glutathione peroxidase (GSH-px) detection kit (A005-3, Nanjing), hydroxyproline (HOP) assay kit (A030-2-1, Nanjing), CTX-1 detection kit (H287, Nanjing), osteocalcin (or Bone GLA protein, BGP) assay kit (H152, Nanjing) and parathyroid hormone (PTH) assay kit (H207, Nanjing) were all purchased from Nanjing Jiancheng Bioengineering Institute (www.njjcbio.com). Rat IL-6 Immunoassay (R6000B, Minneapolis) and rat TNF-α Immunoassay (RTA00, Minneapolis) were obtained from R&D Company.

### Animals Groupings and Experiment Procedure

**Figure 1:**
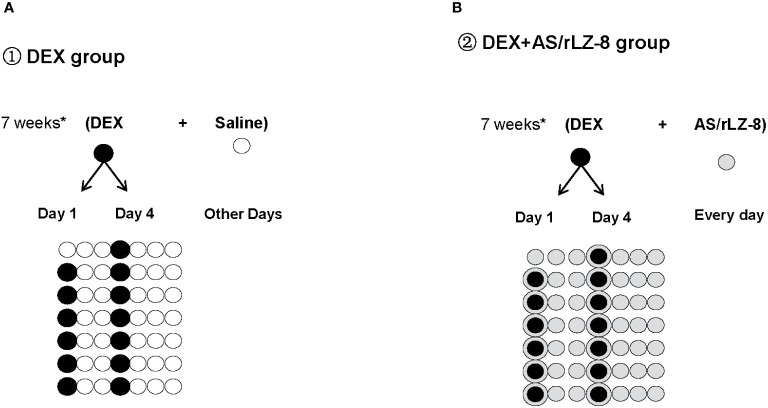
Schematic diagram of the experimental procedure. **(A)** DEX group: White circles (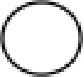) represent days when rats received 2.45 ml/kg BW saline *via* IP injection during the 7 weeks of GIOP modeling. Black circles (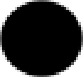) represent days when rats received 2.5 mg/kg BW DEX *via* IM injection at the first and fourth days of each week for 7 weeks. **(B)** DEX+AS/rLZ-8 group: Grey circles (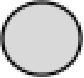) represent days when rats received 28.0 μg/kg, 56.0 μg/kg and112.0 μg/kg BW rLZ-8 *via* IP injection or 1 mg/kg BW AS *via* intragastric administration. Circled bullets (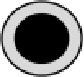) represent days when rats received DEX as well as rLZ-8 or AS.

Wistar rats used in this study were all obtained from Animal Center of Jilin University and maintained following Specific Pathogen Free (SPF) standard with 21 ± 2 °C temperature and 12 h light/dark cycle in the same room. Sixty female rats (6 weeks old,weighting 180 ± 20 g, License No. SCXK (Jing) 2009-0004) were prepared for the testing of potential protective effects of rLZ-8 against GIOP and were evenly randomized divided into six groups with 10 in each group, blank control group (saline group), GIOP model group (DEX group), positive control group (DEX + AS group), low dose rLZ-8 group (DEX + 28.0 μg/kg BW rLZ-8), medium dose rLZ-8 group (DEX + 56.0 μg/kg BW rLZ-8) and high dose rLZ-8 group (DEX + 112.0 μg/kg BW rLZ-8). GIOP rats were generated through intramuscular (IM) injection of DEX at 2.5 mg/kg bodyweight (BW), twice each week for 7 weeks (**Figure 1**).

1. Saline group: rats receive 2.45 ml/kg BW saline at 8:00 and 16:00 *via* intraperitoneal (IP) injection each day. Starting from day 4, rats receive 2.5 ml/kg saline at 8:00 *via* IM injection at the first and fourth days of each week for 7 weeks.
2. DEX group: rats receive 2.45 ml/kg BW saline twice a day each as described in saline group. From day 4, administrate 2.5 mg/kg DEX to each rat at 8:00 *via* IM injection at the first and fourth days of each week for 7 weeks (**Figure 1A**, 13 black circles in days 1 and 4 of the 7 weeks, excepting day 1 for the first week of the experiment).
3. DEX + AS group: rats receive 1 mg/kg BW AS at 8:00 *via* intragastric administration each day for 7 weeks. Dosing for DEX is the same as described in DEX group.
4. DEX + rLZ-8 group: rats received 2.45 ml/kg BW different concentration of rLZ-8 solutions twice a day *via* IP injection. The concentrations of rLZ-8 solution used in low dose rLZ-8 group (DEX + 28.0 μg/kg BW rLZ-8), medium dose rLZ-8 group (DEX + 56.0 μg/kg BW rLZ-8) and high dose rLZ-8 group (DEX + 112.0 μg/kg BW rLZ-8) are 11.43 μg/ml, 22.87 μg/ml and 45.74 μg/ml respectively. Dosing for DEX is the same as described in DEX group.

For the test of reversal capacity of rLZ-8 against existing GIOP, 30 male rats weighting 275 ± 25 g (License No. SCXK (Ji) 2007-0003) were randomized divided into three groups with 10 animals in each group, blank control group (saline group), GIOP model group (DEX group) and rLZ-8 reversal group (DEX + 112.0 μg/kg BW rLZ-8). For the DEX + rLZ-8 group, DEX was given within the first 7 weeks and rLZ-8 administration starting from days 50 to 70 (3 weeks).

On the final day of each experiment, all rats were fasted and separately placed in a metabolic cage for 24 h to collect urine sample. Twenty four hours after the last dosing, all rats were anesthetized with 10% chloral hydrate. Femur and serum samples were obtained during autopsy, and all the samples were stored at −80 °C until further assessment.

### Measurement of Femur Bone Mineral

The bone density of rats’ femur bones was examined by single photo bone mineral density analyzer (HH6005, Hehai, Beijing). The scanning results processed by the software of HH6005 could be output as bone mineral content (BMC, g/cm, mineral amount/scanning length) or bone mineral density (BMD, g/cm^2^, mineral amount/scanning area).

### HE Staining and Bone Histomorphometry

Fix the femur in 4% formaldehyde solution for 72 h at room temperature. Incubate the fixed femur in decalcifying solution for 1 month at room temperature. After paraffin embedding, the specimen is sectioned in 5 μm slices. The sections were stained with hematoxylin and eosin (HE staining) and observed under light microscope (Osteoplan II; Carl Zeiss, NY). Bone histomorphometry is quantified with trabecular area index calculated as trabecular volume in groups normalized with that in saline group. Bone marrow adipocytes is quantified by adipocytes number index calculated as adopocytes number in fixed area of each group/adopocytes number in fixed area of saline group. The area was measure with Adobe Photoshop CS4 extended (version 11.0.1).

### Western Blot

One hundred milligrams of bone tissue was pulverized vigorously with 0.4 ml lysis buffer (20 mM Tris, 150 mM NaCl, 1% Triton X-100) for 30 min. It was centrifuged at 12,000 rpm * 10 min at 4 °C and kept the supernatant. β-actin was chosen as internal control for both anti-OPG and anti-RANKL. Detection of the band was based on enhanced chemiluminescence method (PW30301S, MonPro™ ECL Star Substrate, Monad Biotech Co., Ltd, Wuhan, China). Image J was applied to quantify the intensity of each band. Intensities ratio of OPG/β-actin, RANKL/β-actin and OPG/RANKL were calculated and plotted into graphs respectively.

### Assays

Concentrations of Pi, Ca^2+^, HOP, ALP, TRACP5b, SOD, MDA, GSH-px were all determined by Beckman Coulter (AU680) using commercialized kits listed in *Materials*. Serum levels of IL-1β, IL-6, TNF-α, PTH, Osteocalcin, CT, E_2_ and T were determined using ELISA by commercialized kits. Creatinine concentration in urine was measured with Beckman Coulter (AU680). The value of urinary P, HOP and Ca^2+^ were all corrected by creatinine concentration. Assays were performed according to the instruction manual. The coefficients of variation were less than 10% for all assays.

### Statistical Analysis

SPSS 25.0 package (SPSS Inc, Chicago, IL) was used for data analysis. Data are presented as the means ± standard deviation (SD). ANOVA and LSD were performed for the comparisons between two groups. *P* values less than 0.05 were considered significant.

## Results

### rLZ-8 Could Prevent Bone Mass Loss in Femur of GIOP Rats

**Figure 2:**
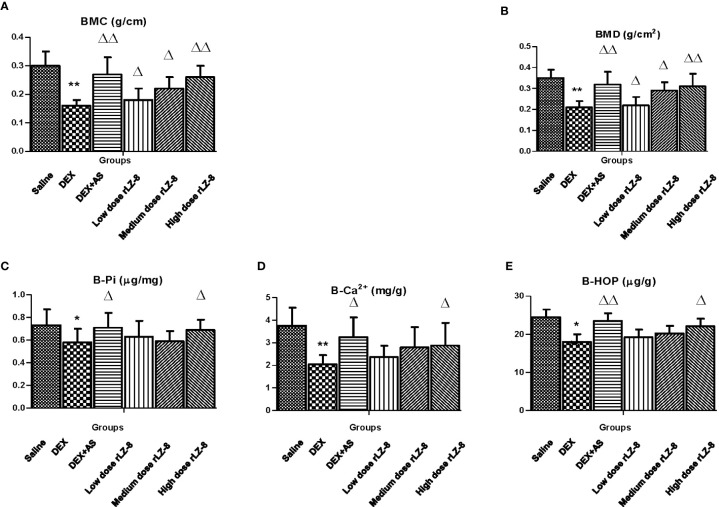
Effects of rLZ-8 on bone density reduction of femur caused by DEX. Bone density of each group was presented as BMC **(A)** and BMD **(B)**. BMC in DEX groups decreased significantly compared to saline groups (^**^P < 0.01). AS and different dose of rLZ-8 co-treatment with DEX increased the BMC compared to the DEX group. BMD has the same trend as BMC. Each groups use five independent slices. Concentration of phosphorus **(C)**, calcium **(D)** and hydroxyproline **(E)** in femurs were measured in each group. ^*^P < 0.05 versus saline group; ^**^ *P <* 0.01 versus saline group; ^△^*P <* 0.05 versus DEX group; ^△△^*P <* 0.01 versus DEX group.

To quantify the bone density, we analyzed the BMC and BMD value of the right femurs from each rats (**Figures 2A, B**). Physical parameters of the femurs including length, diameter and weight were described in **Table 1**. Comparing to saline group, five other groups with IM injection of DEX all decreased significantly in BMC and BMD (all *P* < 0.05). Comparing to the DEX group, positive control group (DEX + AS) and three different dose rLZ-8 groups have shown obvious increase in BMC and BMD (*P* < 0.05), suggesting rLZ-8 protected against bone density decrease in GIOP rats and high dose rLZ-8 shown similar effect with AS. Comparing the effects among different doses of rLZ-8, there is an obvious difference among groups, indicating the partial dose dependent effect of rLZ-8.To verify the results above, we also measured the concentrations of Pi, Ca^2+^, and HOP in bones (B-Pi, B-Ca^2+^, and B-HOP). Comparing to the saline group, five other groups all decreased significantly group in B-Pi, B-Ca^2+^, and B-HOP (*P < 0.05, **P < 0.01 and *P < 0.05 respectively). As shown in **Figures 2C–E**, high dose rLZ-8 significantly improved the B-Pi, B-Ca^2+^, and B-HOP values comparing to the DEX group (^△^*P <* 0.05).

**Table 1:** Description of 60 right femurs each group.

Grouping (n = 10)	Length of Femur (mm)	Diameter of Femur (mm)	Weight of Femur (g)
**Saline**	33.62 ± 2.27	3.51 ± 0.23	0.75 ± 0.12
**DEX**	33.59 ± 1.39	3.35 ± 0.51	0.72 ± 0.07
**DEX + AS**	33.82 ± 0.82	3.41 ± 0.32	0.76 ± 0.11
**28.0 μg/kg rLZ-8**	33.91 ± 1.42	3.52 ± 0.45	0.73 ± 0.21
**56.0 μg/kg rLZ-8**	33.72 ± 1.09	3.57 ± 0.28 *	0.72 ± 0.15
**112.0 μg/kg rLZ-8**	34.01 ± 1.20*	3.64 ± 0.46 **	0.77 ± 0.09*

Physical parameters of femurs, including length, diameter and weight in each group have been measured. The three parameters in DEX group decreased to a certain extent, but without significant difference compared with the saline group (P > 0.05). AS improved the three parameter but without significant difference (P > 0.05). In the medium dose of rLZ-8 group, the diameter of femur show statistically significant difference comparing to the DEX group (*P < 0.05). In the high dose of rLZ-8 group, the length, diameter and weight were all significantly improved (*P < 0.05, **P < 0.01 and *P < 0.05 respectively).

### rLZ-8 Could Prevent Structural Deterioration in Femurs of GIOP Rats

**Figure 3:**
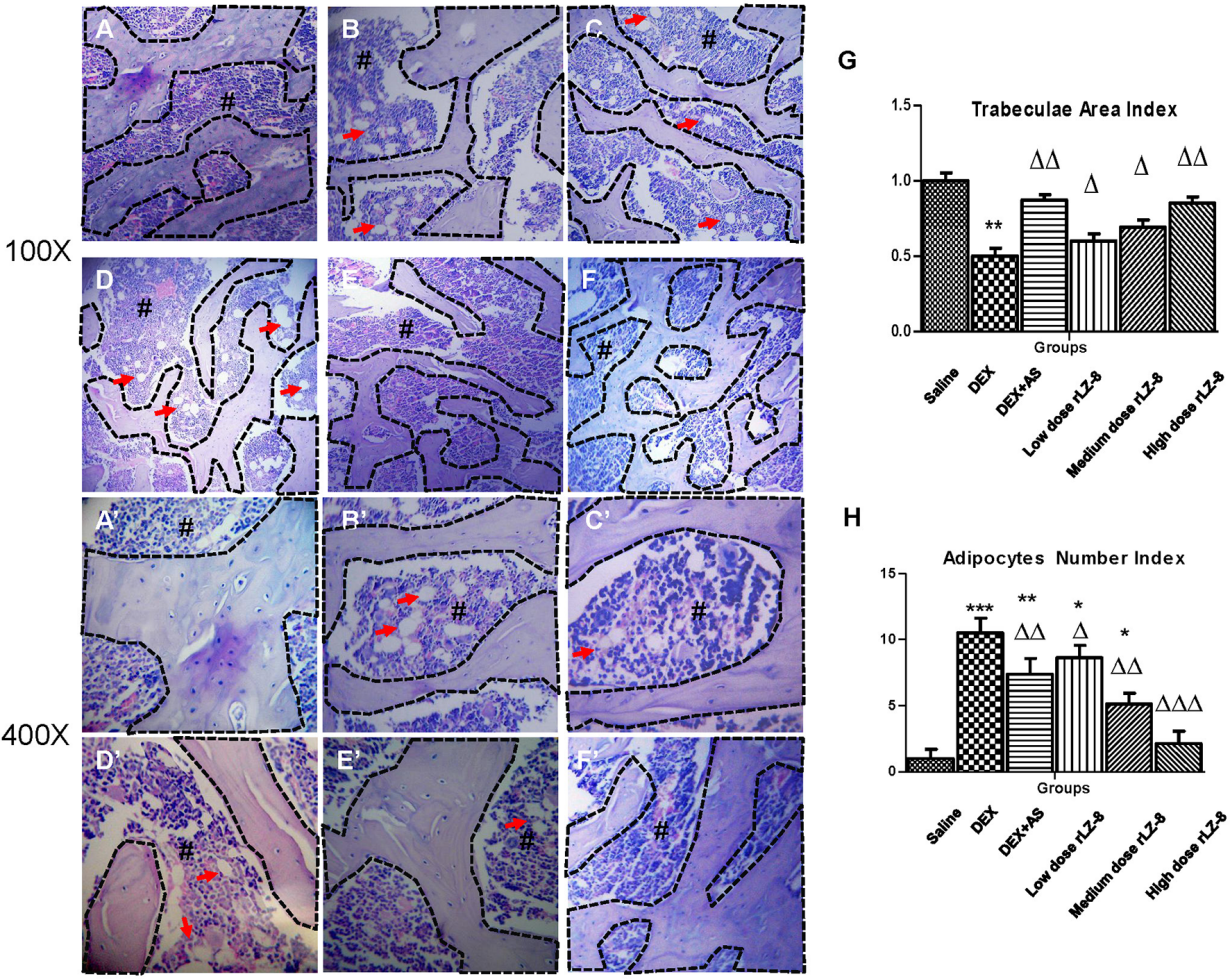
Protective effect of rLZ-8 on bone histomorphometry change caused by DEX. Groups **(A–F)** are the representative HE staining graphs taken at 100-fold magnification of blank control group **(A)**, GIOP model group **(B)**, positive control group (**C**, DEX + AS), rLZ-8 low dose group **(D)**, DEX + 28.0 μg/kg BW rLZ-8), rLZ-8 medium dose group (**E**, DEX + 56.0 μg/kg BW rLZ-8) and rLZ-8 high dose group **(F)**, DEX + 112.0 μg/kg BW rLZ-8). Graphs **(A–F)** are taken at 400-fold magnification. The area marked # with deeper staining is the bone marrow region. Cells labeled by arrows are bone marrow adipocytes. The area bounded by a dotted line represents the bone trabeculae region. **(G)**: trabeculae area index (trabeculae area in each group/area in the saline group) of the six groups. **(H)**: Adipocytes number index (adopocytes number in fixed area of each group/adopocytes number in fixed area of saline group) of the six groups. Each groups use five independent slices (*P < 0.05, **P < 0.01 and ***P < 0.005 versus saline group; ^△^*P <* 0.05, ^△△^*P <* 0.01, and ^△△△^*P <* 0.005 versus DEX group).

To examine the structural deterioration in femurs of GIOP rats, we performed H&E staining (**Figures 3A–F****’**). In the saline group, the trabeculae of rats were smooth and closely connected exhibiting a reticular structure. Comparing to the saline group, the GIOP model group exhibited reduction in trabeculae volume and abnormal morphologies like shape distortion or fracture. To quantify the trabeculae volume, we calculated the trabeculae area index (described in *HE Staining and Bone Histomorphometry*). As shown in **Figure 3G**, different doses of rLZ-8 could all increase the trabeculae area index significantly comparing to the DEX group. Besides the defects in trabeculae, the bone marrow cavity was also significantly enlarged with more light stained area composed by adipocytes. To quantify the number change of adipocytes, we calculated the adipocytes number index (described in *HE Staining and Bone Histomorphometry*). Comparing to the saline group, adipocytes number in DEX group increased significantly (*P <* 0.005) in DEX group (**Figure 3H**). Comparing to the DEX group, positive control group (DEX + AS) and three different dose rLZ-8 groups have shown obvious decrease in adipocytes (all *P* < 0.05). Notably, adipocytes number in the high dose rLZ-8 group were comparable to saline group (*P >*0.05).

### Toxicity Study of rLZ-8 *via* Body Weight and Organ Coefficient Analysis

**Figure 4:**
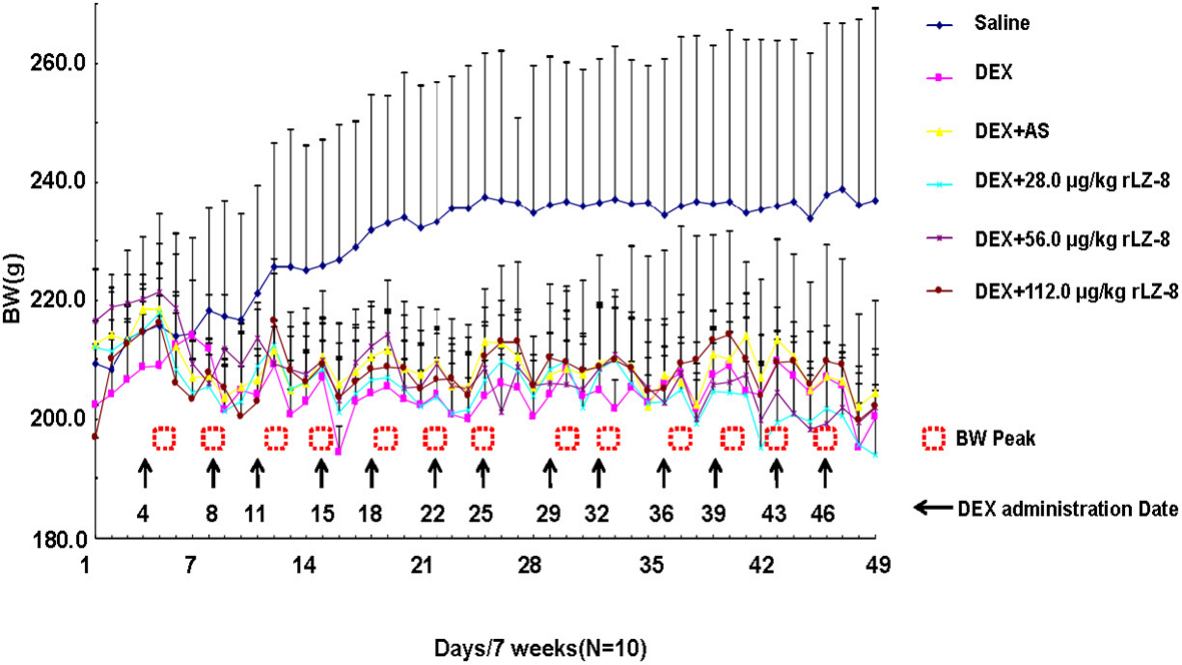
Body weight change of the female Wistar rats within 7 weeks. The initial BWs of female rats in each group are similar and BWs of each rats was recorded daily for 49 days. Six curves with different color represent the BWs change in the six groups respectively. Arrows stand for the date receiving DEX and dashed squares stand for the peaks of BW. (BWs were presented as mean ± standard deviation).

To identify potentially harmful effects of rLZ-8, BWs and organ coefficient (organ weight to body weight ratios) have been investigated (**Figure 4** and **Table 2**). In the daily feeding, the dietary intake of the rats after the IM injection of DEX was reduced, accompanied with severe hair loss. BWs of each rat were recorded every day and plotted into graph. BWs in saline group increased steadily and are higher than five groups during the whole process. BWs in other five groups would decrease when received IM injection of DEX (arrows in **Figure 4**) and increase gradually to the level before injection (dashed square). Until the end of the experiment, there was no significant difference in the body weight of the rats in each group. As shown in **Table 2**, compared with the saline group, coefficient in heart, liver (*P <* 0.05), spleen (*P <* 0.05), lung, and kidney (*P < *0.01) of the model group all increased.

**Table 2:** Results of Various Organ Coefficient in Rats (n = 10, g/100 g BW).

Groupings	Organ Coefficient (mg/10 g, $\bar{x}$ ± s)					
	Heart	Liver	Lungs	Kidney	Thymus	Spleen
**Saline**	0.36 ± 0.04	3.31 ± 0.33	0.64 ± 0.12	0.65 ± 0.03	0.16 ± 0.05	0.26 ± 0.04
**DEX**	0.39 ± 0.04	3.82 ± 0.29^*^	0.67 ± 0.08	0.77 ± 0.05^**^	0.14 ± 0.03	0.18 ± 0.02^*^
**DEX + AS**	0.40 ± 0.02	3.43 ± 0.35	0.62 ± 0.09	0.77 ± 0.04^**^	0.12 ± 0.02	0.20 ± 0.02^*^
**DEX + rLZ-8 28.0 μg/kg**	0.45 ± 0.06*	3.45 ± 0.33	0.60 ± 0.08	0.78 ± 0.06^**^	0.13 ± 0.03	0.19 ± 0.04^*^
**DEX + rLZ-8 56.0 μg/kg**	0.40 ± 0.04	3.30 ± 0.19	0.57 ± 0.06	0.72 ± 0.05^*^	0.14 ± 0.05	0.21 ± 0.03^*^
**DEX + rLZ-8 112.0 μg/kg**	0.39 ± 0.04	3.45 ± 0.26	0.56 ± 0.05	0.76 ± 0.05^**^	0.15 ± 0.03	0.20 ± 0.04^*^

Organ coefficient is calculated ratio of organ weight to body weight after fasting. Comparing with saline group, the coefficient of heart, liver, spleen, lung, and kidney of the model group increased to a certain extent, and the thymus coefficient decreased. * stands for P < 0.05，**P < 0.01.

### Influence of rLZ-8 on Biomarkers of GIOP in Serum and Urine

**Figure 5:**
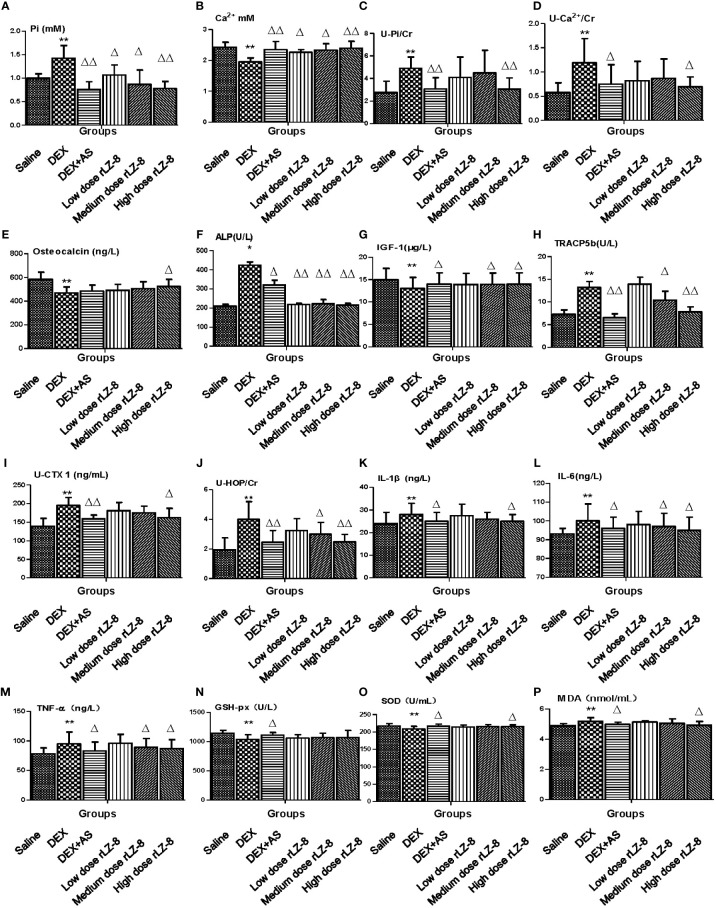
Effect of rLZ-8 on level of bone metabolic markers in GIOP rat’s serum and urine. **(A)** Serum phosphorus (mM); **(B)** Serum calcium (mM); **(C)** Urinary phosphorus/Cr (mM/mg); **(D)** Urinary calcium/Cr (mM/mg); **(E)** Serum osteocalcin (ng/L); **(F)** Serum ALP (U/L); **(G)** Serum IGF-1 (μg/L); **(H)** Serum TRACP5b (U/L); **(I)** Urinary CTX1/Cr (ng/mL * mg); **(J)** Urinary HOP/Cr (mM/mg); **(K)** IL-1β (ng/L); **(L)** IL-6 (ng/L); **(M)** TNF-α(ng/L); **(N)** GSH-px (U/L); **(O)** SOD (U/mL); **(P)** MDA (nmol/L). Compared with the blank control group, * stands for *P <* 0.05 and ** stands for *P <* 0.01; Compared with the model group, △ stands for *P <* 0.05 and △△ stands for *P <* 0.01.

To verify the results obtained from bone analysis, we examined several different types of biomarkers in serum or urine, including minerals (**Figures 5A–D**, serum Pi, serum Ca^2+^, urinary Pi and urinary Ca^2+^), bone formation markers (**Figures 5E–G**, serum osteocalcin, serum ALP and serum IGF-1), bone resorption markers (**Figures 5H–J**, serum TRACP5b, urinary CTX-1 and urinary HOP), cytokines(**Figures 5K–M**, serum IL-1β, serum IL-6 and serum TNF-α), oxidative stress indicators (**Figures 5N–P**, serum GSH-px, serum SOD and serum MDA), hormone molecules (**Supplementary Figures 1A–D**, serum CT, serum PTH, serum testosterone and serum estradiol). Comparing to DEX group, rLZ-8 groups had reduced serum Pi level (**Figure 5A**), increased serum Ca^2+^ level (**Figure 5B**), reduced urinary Pi level (**Figure 5C**), reduced urinary Ca^2+^ level (**Figure 5D**),increased serum osteocalcin level (**Figure 5E**), reduced serum ALP activity (**Figure 5F**), increase serum IGF-1 level (**Figure 5G**), reduced serum TRACP5b level (**Figure 5H**), reduced urinary CTX-1 level (**Figure 5I**), reduced urinary HOP level (**Figure 5J**), reduced serum cytokines (**Figures 5K–M**), reduced SOD activity (**Figure 5O**), reduced MDA level (**Figure 5P**), reduced serum CT level (**Supplementary Figure 1A**) and reduced PTH level (**Supplementary Figure 1B**).

### Influence of rLZ-8 on Protein Expression Level of OPG and RANKL in Bone Tissue

**Figure 6:**
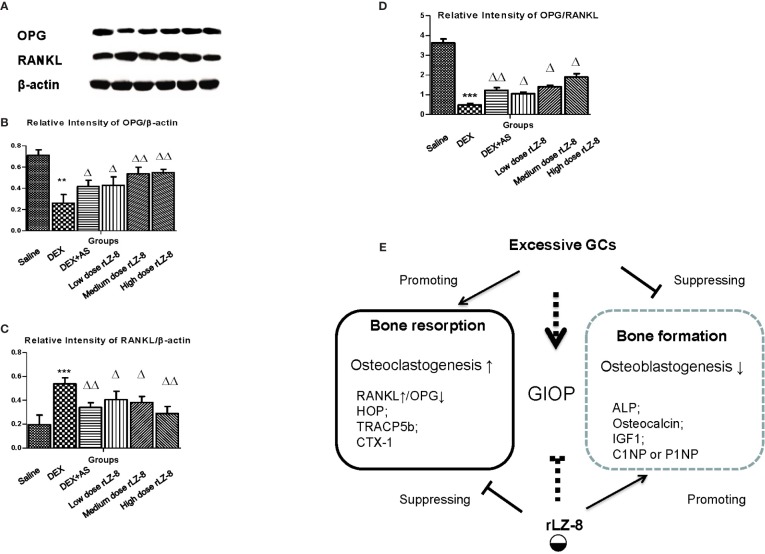
Influence of rLZ-8 on protein expression level of OPG and RANKL in bone tissue. **(A)** Bands of anti-OPG, anti-RANKL, and anti-β-actin in rat femur bone by western blot. From the left to the right, the bands are from blank control group, GIOP model group, positive control group, low dose of rLZ-8 group, medium dose of rLZ-8 group and high dose of rLZ-8 group respectively. **(B)** Relative expression level of OPG/β-actin in each group. **(C)** Relative expression level of RANKL/β-actin in each group. **(D)** Relative expression level of OPG/RANKL in each group. **(E)** Working model of rLZ-8 on GIOP rats. rLZ-8 influences both the bone formation and bone resorption processes. Compared with the blank control group, ** stands for *P <* 0.01 and *** stands for *P <* 0.005; Compared with the model group, △ stands for *P <* 0.05 and △△ stands for *P <* 0.01.

To investigate whether the mechanism of rLZ-8 preventing bone loss was RANK/RANKL/OPG dependent or not, we analyzed the relative expression level of OPG and RANK in femurs *via* western blot. As shown in **Figure 6A**, RANKL and OPG could be clearly detected in the saline group, GIOP model group, positive control group, and three different dose rLZ-8 groups. Comparing to the saline group, relative expression of OPG protein level decreased (OPG/β-actin, **Figure 6B**) and RANKL protein level increased (RANKL/β-actin, **Figure 6C**). As an indicator for OPG/RANKL balance (**Figure 6D**), we found OPG/RANKL ratio varied from groups, which is 3.62- (saline group), 0.49- (DEX group), 2.44- (DEX + AS groups), 1.00- (DEX + 28.0 μg/kg BW rLZ-8), 1.58- (DEX + 56.0 μg/kg BW rLZ-8), and 1.81- (DEX + 112.0 μg/kg BW rLZ-8) fold, respectively (*P <* 0.05).

### rLZ-8 Could Also Reverse the Bone Loss in GIOP Rats

**Figure 7:**
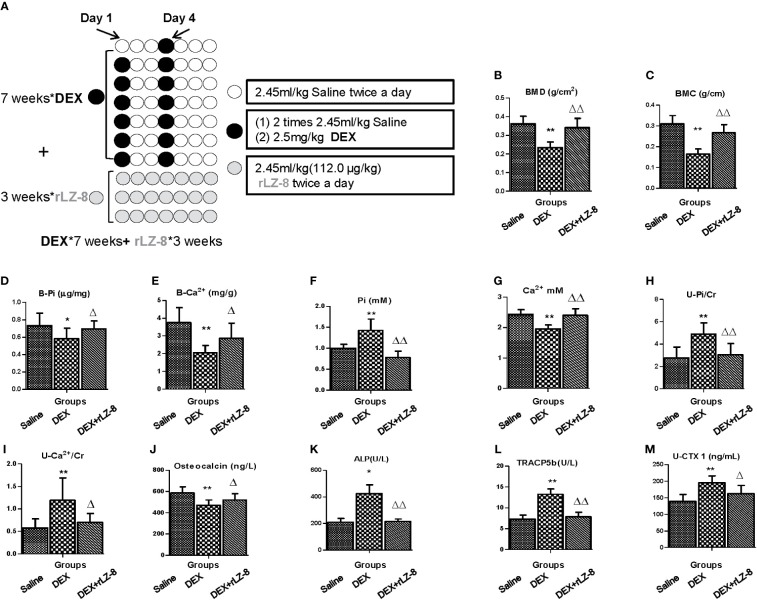
rLZ-8 could reverse bone loss in GIOP rats. **(A)** Schematic diagram of experimental procedure for testing the reversal effects of rLZ-8 on bone mass of GIOP rats. **(B)** BMD (g/cm^2^); **(C)** BMC (g/cm); **(D)** Phosphorus in bone (μg/mg); **(E)** Calcium in bone (mg/g); **(F)** Serum phosphorus (mM); **(G)** Serum calcium (mM); **(H)** Urinary phosphorus/Cr (mM/mg); **(I)** Urinary calcium/Cr (mM/mg); **(J)** Serum osteocalcin (ng/L); **(K)** Serum ALP (U/L); **(L)** Serum TRACP5b (U/L); **(M)** Urinary CTX1/Cr (ng/ml * mg). Compared with the blank control group, * stands for *P <* 0.05 and ** stands for *P <* 0.01; Compared with the model group, △ stands for *P < *0.05 and △△ stands for *P <* 0.01.

To test the potential reversal effect of rLZ-8 on successfully generated GIOP rats, the rLZ-8 administration should start from Day 50 when the bone loss already occurred. Following the experiment procedures described in **Figure 7A**, BMC, BMD, Pi level in bone and Ca^2+^ level in bone (**Figures 7B–E**) were all elevated significantly by 112.0 μg/kg BW rLZ-8(*P* < 0.05), suggesting rLZ-8 could also reverse the bone density loss after the establish of GIOP model. In addition, we also examine levels of serum phosphorus, serum calcium, urinary phosphorus/Cr, urinary calcium/Cr, serum osteocalcin (ng/l), serum ALP, serum TRACP5b and urinary CTX1/Cr (ng/ml * mg) (**Figures 7F–M**), which is consistent with the results of protective assay.

## Discussions

*G. lucidum*, as a traditional Chinese medicine with more than 2,400 years old history, has long been acclaimed as an effective immune stimulant in promoting health (Cor et al., 2018). In 2009, ethanol extract of *G. lucidum* was first found to prevent bone loss in Ovx rats, which inspired investigators to connect *G. lucidum* and anti-osteoporosis together (Miyamoto et al., 2009). Until now, two compounds like ganoderic acid DM and ganomycin I have been discovered as active components in regulating osteoclastogenesis which provided anti-osteoporosis capacity to *G. lucidum* (Miyamoto et al., 2009; Tran et al., 2018). Besides the small molecules mentioned above, LZ-8, first FIP family protein isolated from *G. lucidum* (Kino et al., 1989), functioned as immune modulator in immune cell and cancer cell (Haak-Frendscho et al., 1993; Huang et al., 2009). Accumulating evidences in osteoimmunology field suggested that immune modulation was a promising anti-osteoporosis strategy (Tsukasaki and Takayanagi, 2019; Guder et al., 2020). Therefore, we hypothesized that LZ-8 might affect osteoporosis through immune modulation. In this study, considering the convenience of the present experiment and future potential clinical use, we used rLZ-8 purified from yeast recombinant expression system with obvious advantages in yield and repeatability over native LZ-8. Considering long term GCs could imbalance the immune regulation, we chose GIOP rats as animal model for rLZ-8 study.

Clinically, patients at high risk of GIOP and patients already developed GIOP had different demands. For the patients had to start GCs therapy but without osteoporosis symptom yet, drugs with protective or preventive capacity would help. For the GIOP patients, drugs with therapeutic capacity or bone loss reversal capacity were required. We addressed this view by setting different timing strategies of rLZ-8 administration. For investigate the potential preventive or protective effect, rLZ-8 should be administrated before the GIOP modeling. Referring to studies with the similar intention, we co-treated the rLZ-8 with DEX as previously described (Liu et al., 2011; Tran et al., 2018). For investigate the potential therapeutic or reversal effect, we started to treat rLZ-8 to rats after the GIOP modeling was done.

Bone mass loss is the key feature for osteoporosis and BMD is regarded as the WHO standard for diagnosis of osteoporosis. However, the poor sensitivity of BMD suggested that multiple biomarker should be used to avoid false negative (Wheater et al., 2013). In our study, to improve the reliability of the data, we also measured B-Pi, B-Ca^2+^, and B-HOP in addition to BMC and BMD (**Figure 2**). Besides the bone mass loss, structural deterioration is another essential characteristic for osteoporosis. In our study, the trabeculae area index and adipocytes number index were developed for the bone histomorphometry study. Together with the shrink of trabeculae area, bone marrow cavity was enlarged and occupied by adipocytes in the DEX group. Bone marrow mesenchymal stem cells (BMSCs) are the common precursor cell to adipocytes and osteoblast cells. Adipocytes accumulation or lipid toxicity is frequently reported in various diseases, including postmenopausal osteoporosis, aging osteoporosis and osteonecrosis (Gillet et al., 2017). Some investigators found that bone marrow adipocytes could suppress osteoblast differentiation through secreting factors like PPARγ, adiponectin or fatty acids (Gillet et al., 2017).

Besides analyzing the bone tissue directly, biomarkers of bone turnover in serum or urine are frequently used to investigate the metabolic status of bone in both bench and clinical (Takahashi et al., 2010; Wheater et al., 2013). The statistical differences between DEX group and rLZ-8 groups in multiple biomarkers in serum and urine, suggested that rLZ-8 could affect multiple biomarkers in various processes. First, rLZ-8 could reset the balance of phosphorus and calcium among bone, serum and urine impaired by excess DEX (**Figures 2D, E** and **5A–D**). Second, rLZ-8 could affect markers of both bone formation and bone resorption. Most of the anti-osteoporosis drugs available are anti-resorptive drugs including bisphosphonates AS used in our study (Rizzoli and Biver, 2015). High dose rLZ-8 performed better in affecting bone formation markers than anti-resorptive drug, AS (**Figures 5E–G**). Third, rLZ-8 could reduce the level of cytokines as well as oxidative stress elevated by excess DEX in rats (**Figures 5K–P**). Fourth, rLZ-8 could also affect the hormone level including CT, PTH and T (**Supplementary Figure 1**). Collectively, rLZ-8 could attenuate bone loss in GIOP rats *via* multiple mechanisms and probably couple anti-resorptive and anabolic benefits in GIOP rats.

Developing drugs from natural products were considered as promising strategies with few side effects (Cor et al., 2018). Meanwhile, the current available synthetic anti-osteoporosis drugs likes bisphosphonates and Denosumab, were limited by the side effects (Bonani et al., 2017). In this study, we analyzed the BW and organ coefficient for the toxicity study. In DEX group, organ coefficient of coefficient in liver (increased, *P <* 0.05), spleen (decreased, *P <* 0.05), and kidney (decreased, *P <* 0.01). However, three different doses of rLZ-8 could alleviate this phenomenon to a certain extent, indicating that rLZ-8 can antagonize the effect of DEX on the corresponding organs to a certain extent. Taken together, rLZ-8 had little toxicity for the animal.

LZ-8 has dual roles in immune modulation. Originally, LZ-8 is regarded as pro-inflammatory factor in activating immune cells T cells or dendrite cells and NF-κB (Haak-Frendscho et al., 1993; Lin et al., 2009; Hsu et al., 2013). Some studies also reported LZ-8 could serve as anti-cancer factors through arresting cell growth, inducing cell death or affecting tumor metastasis (Wu et al., 2011; Liang et al., 2012; Lin and Hsu, 2016). Interestingly, LZ-8 in wound healing of rat liver damage model acts like a anti-inflammatory factor by reducing NF-κB expression level which seems controversial comparing to the activation of NF-κB in immune system (Lin et al., 2014). From the serum level measure, we found rLZ-8 could reduce expression of the serum IL-6 and TNF-α which suggested rLZ-8 might serve as anti-inflammatory factors in treating GIOP. In response to inflammatory cytokine, osteoclast precursors would activate RANKL/RANK pathway for the differentiation of osteoclast (Boyce and Xing, 2008). Meanwhile, our study shows that in the circumstance of GIOP, rLZ-8 could reduce the expression level of RANKL (consistent with NF-κB activity) in bone tissue, which is similar with the wound healing results above. Therefore, we proposed that LZ-8 has dual role in modulating inflammatory reaction, acting as pro-inflammatory factor in immune cells and anti-inflammatory factor in would healing or GIOP (**Figure 6E**).

## Conclusion

In this study, we successfully replicate the DEX induced osteoporosis rats model. Recombinant LZ-8, immune modulator derived from *G. lucidum*, could prevent bone mass loss measured by BMC, BMD, levels of Pi, Ca^2+^ and HOP in femur of GIOP rats. In addition, rLZ-8 could also prevent the bone structural deterioration in of GIOP rats by analyzing the H&E staining of trabeculae in femurs. Through the BWs and organ coefficient analysis, rLZ-8 has little toxicity to the rats. Furthermore, we found rLZ-8 could affect multiple biomarkers of GIOP in serum and urine which verified the results obtained from bone analysis and probably couple anti-resorptive and anabolic benefits in GIOP rats. Further, rLZ-8 could improve osteoclast genesis through down-regulating RANKL and up-regulating OPG which suggest LZ-8 has dual role in modulating inflammatory signaling. Finally, we found that rLZ-8 could also help when administrated after the modeling GIOP rats. Collectively, our results suggested that LZ-8 is the first macromolecules isolated from *G. lucidum* serving as promising anti-osteoporosis drug coupling anti-resorptive and anabolic benefits in GIOP.

## Data Availability Statement

The raw data supporting the conclusions of this article will be made available by the authors, without undue reservation, to any qualified researcher.

## Ethics Statement

The animal study was reviewed and approved by the Animal Ethics Committee of the School of Pharmaceutical Sciences, Jilin University.

## Author Contributions

YY and JP wrote the manuscript. YY, TY, and JP designed the experiments. YY, TY, HT, ZR, QL, JJ, HC, JF, SD, QH, DX, LS, and BS performed the experiments. FS performed the expression and purification of rLZ-8.

## Funding

This research was funded by the Science and Technology Development Project form Jilin Science and Technology Department (2018 0311072YY), and Jilin Province Development and Reform Commission (2014N149). This research was also supported by Jilin University (Grant number 419080600013).

## Conflict of Interest

The authors declare that the research was conducted in the absence of any commercial or financial relationships that could be construed as a potential conflict of interest.

## Supplementary Material

Supplementary Figure 1 Effect of rLZ-8 on level of hormone molecules in GIOP rats serum. (A) Serum CT (ng/L); (B) Serum PTH (pg/mL); (C) Serum testosterone (ng/mL); (D) Serum estradiol (ng/L). Compared with the blank control group, * stands for P < 0.05 and ** stands for P < 0.01; Compared with the model group, △ stands for P < 0.05 and △△ stands for P < 0.01.
