# Supplementary figures and images for: Ganoderma lucidum Immune Modulator Protein rLZ-8 Could Prevent and Reverse Bone Loss in Glucocorticoids-Induced Osteoporosis Rat Model
Source: Front Pharmacol. 2020 May 19;11:731. doi: 10.3389/fphar.2020.00731 (PMC7248554; doi:10.3389/fphar.2020.00731)

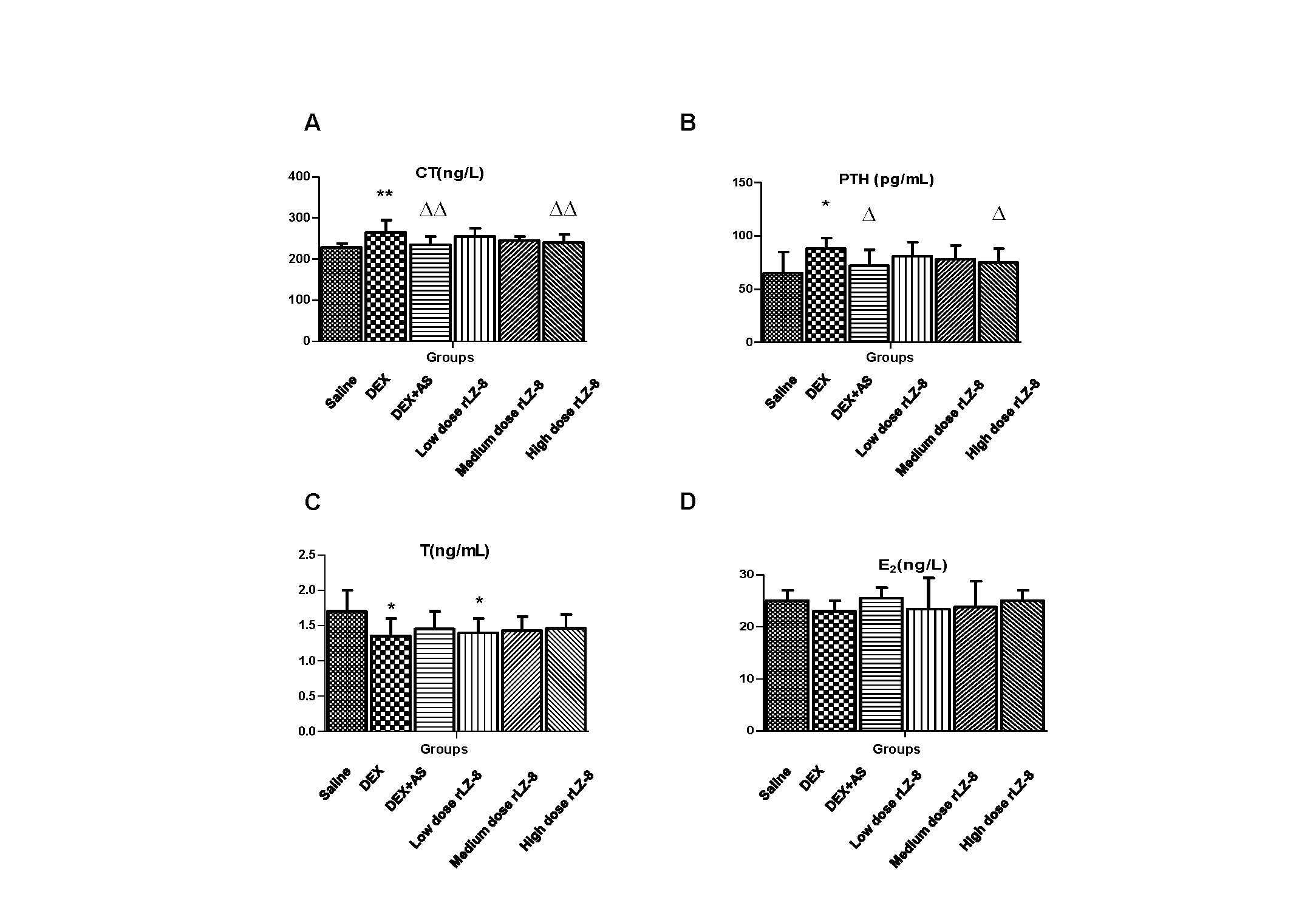

Supplement: Supplementary Figure 1 — Effect of rLZ-8 on level of hormone molecules in GIOP rats serum. (A) Serum CT (ng/L); (B) Serum PTH (pg/mL); (C) Serum testosterone (ng/mL); (D) Serum estradiol (ng/L). Compared with the blank control group, * stands for P < 0.05 and ** stands for P < 0.01; Compared with the model group, △ stands for P < 0.05 and △△ stands for P < 0.01. [file Image_1.jpeg]

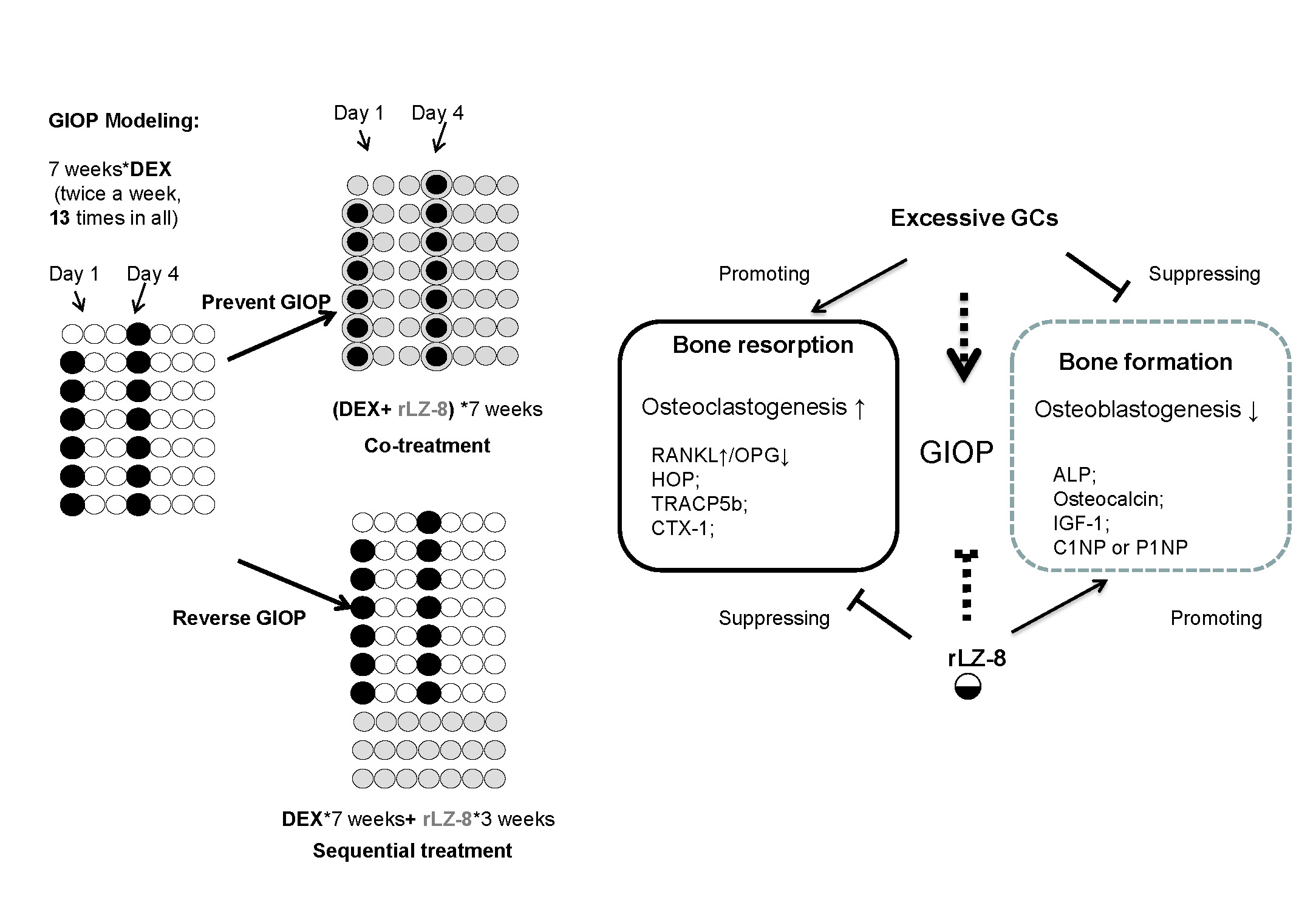

Supplement: Supplementary file 2 [file Image_2.jpeg]
